# An Approximation of the Error Backpropagation Algorithm in a Predictive Coding Network with Local Hebbian Synaptic Plasticity

**DOI:** 10.1162/NECO_a_00949

**Published:** 2017-03-23

**Authors:** James C. R. Whittington, Rafal Bogacz

**Affiliations:** MRC Brain Network Dynamics Unit, University of Oxford, Oxford, OX1 3TH, U.K., and FMRIB Centre, Nuffield Department of Clinical Neurosciences, University of Oxford, John Radcliffe Hospital, Oxford, OX3 9DU, U.K.; MRC Brain Network Dynamics Unit, University of Oxford, Oxford OX1 3TH, U.K., and Nuffield Department of Clinical Neurosciences, University of Oxford, John Radcliffe Hospital, Oxford OX3 9DU, U.K.

## Abstract

To efficiently learn from feedback, cortical networks need to update synaptic weights on multiple levels of cortical hierarchy. An effective and well-known algorithm for computing such changes in synaptic weights is the error backpropagation algorithm. However, in this algorithm, the change in synaptic weights is a complex function of weights and activities of neurons not directly connected with the synapse being modified, whereas the changes in biological synapses are determined only by the activity of presynaptic and postsynaptic neurons. Several models have been proposed that approximate the backpropagation algorithm with local synaptic plasticity, but these models require complex external control over the network or relatively complex plasticity rules. Here we show that a network developed in the predictive coding framework can efficiently perform supervised learning fully autonomously, employing only simple local Hebbian plasticity. Furthermore, for certain parameters, the weight change in the predictive coding model converges to that of the backpropagation algorithm. This suggests that it is possible for cortical networks with simple Hebbian synaptic plasticity to implement efficient learning algorithms in which synapses in areas on multiple levels of hierarchy are modified to minimize the error on the output.

## 1 Introduction

Efficiently learning from feedback often requires changes in synaptic weights in many cortical areas. For example, when a child learns sounds associated with letters, after receiving feedback from a parent, the synaptic weights need to be modified not only in auditory areas but also in associative and visual areas. An effective algorithm for supervised learning of desired associations between inputs and outputs in networks with hierarchical organization is the error backpropagation algorithm (Rumelhart, Hinton, & Williams, 1986). Artificial neural networks (ANNs) employing backpropagation have been used extensively in machine learning (LeCun et al., 1989; Chauvin & Rumelhart, 1995; Bogacz, Markowska-Kaczmar, & Kozik, 1999) and have become particularly popular recently, with the newer deep networks having some spectacular results, now able to equal and outperform humans in many tasks (Krizhevsky, Sutskever, & Hinton, 2012; Hinton et al., 2012). Furthermore, models employing the backpropagation algorithm have been successfully used to describe learning in the real brain during various cognitive tasks (Seidenberg & McClelland, 1989; McClelland, McNaughton, & O’Reilly, 1995; Plaut, McClelland, Seidenberg, & Patterson, 1996).

However, it has not been known if natural neural networks could employ an algorithm analogous to the backpropagation used in ANNs. In ANNs, the change in each synaptic weight during learning is calculated by a computer as a complex, global function of activities and weights of many neurons (often not connected with the synapse being modified). In the brain, however, the network must perform its learning algorithm locally, on its own without external influence, and the change in each synaptic weight must depend on just the activity of presynaptic and postsynaptic neurons. This led to a common view of the biological implausibility of this algorithm (Crick, 1989)—for example: “Despite the apparent simplicity and elegance of the back-propagation learning rule, it seems quite implausible that something like equations […] are computed in the cortex” (O’Reilly & Munakata, 2000, p. 162).

Several researchers aimed at developing biologically plausible algorithms for supervised learning in multilayer neural networks. However, the biological plausibility was understood in different ways by different researchers. Thus, to help evaluate the existing models, we define the criteria we wish a learning model to satisfy, and we consider the existing models within these criteria:

1. *Local computation.* A neuron performs computation only on the basis of the inputs it receives from other neurons weighted by the strengths of its synaptic connections.
2. *Local plasticity.* The amount of synaptic weight modification is dependent on only the activity of the two neurons the synapse connects (and possibly a neuromodulator).
3. *Minimal external control.* The neurons perform the computation autonomously with as little external control routing information in different ways at different times as possible.
4. *Plausible architecture.* The connectivity patterns in the model should be consistent with basic constraints of connectivity in neocortex.

The models proposed for supervised learning in biological multilayer neural networks can be divided in two classes. Models in the first class assume that neurons (Barto & Jordan, 1987; Mazzoni, Andersen, & Jordan, 1991; Williams, 1992) or synapses (Unnikrishnan & Venugopal, 1994; Seung, 2003) behave stochastically and receive a global signal describing the error on the output (e.g., via a neuromodulator). If the error is reduced, the weights are modified to make the produced activity more likely. Many of these models satisfy the above criteria, but they do not directly approximate the backpropagation algorithm, and it has been pointed out that under certain conditions, their learning is slow and scales poorly with network size (Werfel, Xiew, & Seung, 2005). The models in the second class explicitly approximate the backpropagation algorithm (O’Reilly, 1998; Lillicrap, Cownden, Tweed, & Akerman, 2016; Balduzzi, Vanchinathan, & Buhmann, 2014; Bengio, 2014; Bengio, Lee, Bornschein, & Lin, 2015; Scellier & Bengio, 2016), and we will compare them in detail in section 4.

Here we show how the backpropagation algorithm can be closely approximated in a model that uses a simple local Hebbian plasticity rule. The model we propose is inspired by the predictive coding framework (Rao & Ballard, 1999; Friston, 2003, 2005). This framework is related to the autoencoder framework (Ackley, Hinton, & Sejnowski, 1985; Hinton & McClelland, 1988; Dayan, Hinton, Neal, & Zemel, 1995) in which the GeneRec model (O’Reilly, 1998) and another approximation of backpropagation (Bengio, 2014; Bengio et al., 2015) were developed. In both frameworks, the networks include feedforward and feedback connections between nodes on different levels of hierarchy and learn to predict activity on lower levels from the representation on the higher levels. The predictive coding framework describes a network architecture in which such learning has a particularly simple neural implementation. The distinguishing feature of the predictive coding models is that they include additional nodes encoding the difference between the activity on a given level and that predicted by the higher level, and that these prediction errors are propagated through the network (Rao & Ballard, 1999; Friston, 2005). Patterns of neural activity similar to such prediction errors have been observed during perceptual decision tasks (Summerfield et al., 2006; Summerfield, Trittschuh, Monti, Mesulam, & Egner, 2008). In this letter, we show that when the predictive coding model is used for supervised learning, the prediction error nodes have activity very similar to the error terms in the backpropagation algorithm. Therefore, the weight changes required by the backpropagation algorithm can be closely approximated with simple Hebbian plasticity of connections in the predictive coding networks.

In the next section, we review backpropagation in ANNs. Then we describe a network inspired by the predictive coding model in which the weight update rules approximate those of conventional backpropagation. We point out that for certain architectures and parameters, learning in the proposed model converges to the backpropagation algorithm. We compare the performance of the proposed model and the ANN. Furthermore, we characterize the performance of the predictive coding model in supervised learning for other architectures and parameters and highlight that it allows learning bidirectional associations between inputs and outputs. Finally, we discuss the relationship of this model to previous work.

## 2 Models

While we introduce ANNs and predictive coding below, we use a slightly different notation than in their original description to highlight the correspondence between the variables in the two models. The notation will be introduced in detail as the models are described, but for reference it is summarized in Table 1. To make dimensionality of variables explicit, we denote vectors with a bar (e.g.,$\overset{¯}{x}$). Matlab codes simulating an ANN and the predictive coding network are freely available at the ModelDB repository with access code 218084.

### 2.1 Review of Error Backpropagation

**Figure 1:**
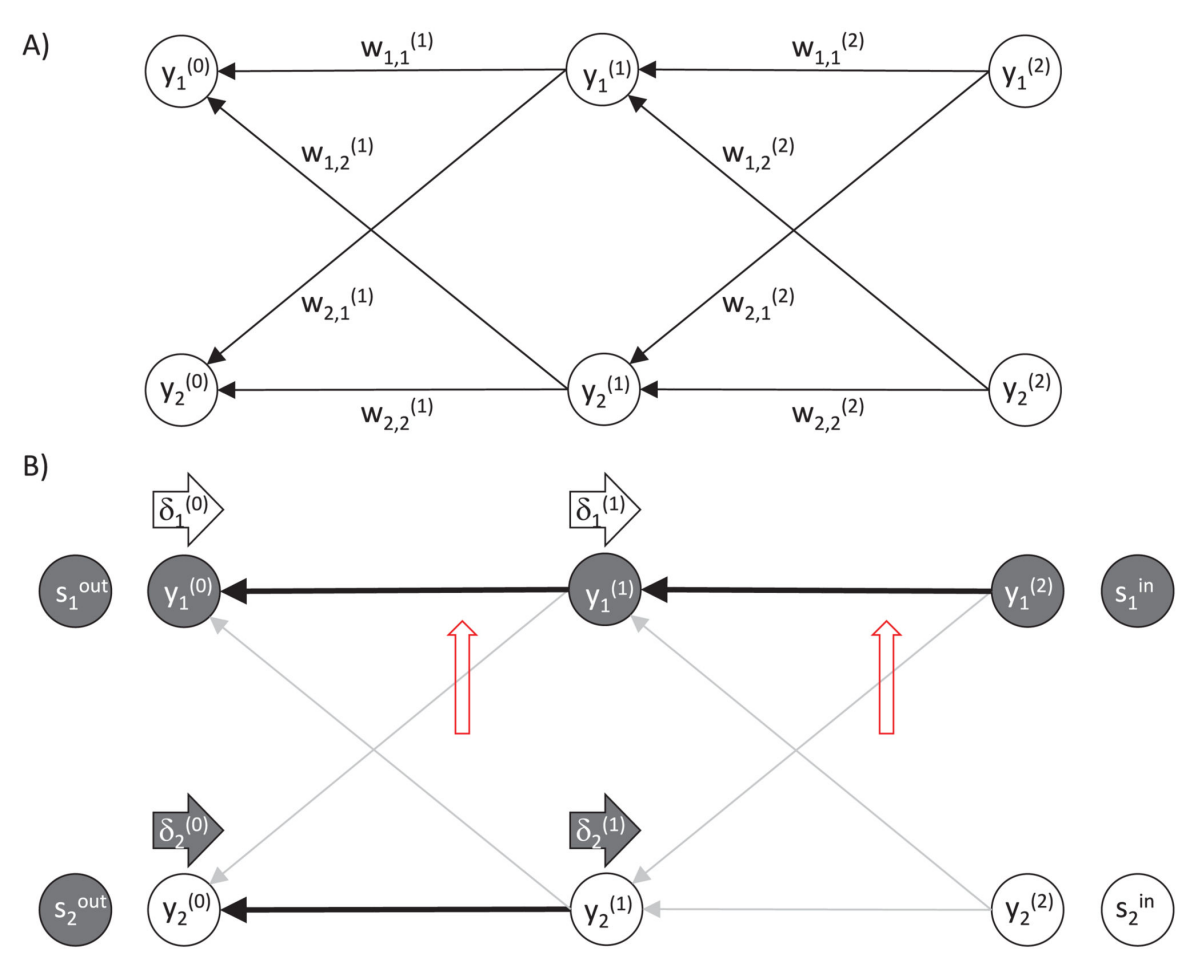
Backpropagation algorithm. (A) Architecture of an ANN. Circles denote nodes, and arrows denote connections. (B) An example of activity and weight changes in an ANN. Thick black arrows between the nodes denote connections with high weights, and thin gray arrows denote the connections with low weights. Filled and open circles denote nodes with higher and lower activity, respectively. Rightward-pointing arrows labeled $\delta_{i}^{\left(l\right)}$ denote error terms, and their darkness indicates how large the errors are. Upward-pointing red arrows indicate the weights that would most increase according to the backpropagation algorithm.

ANNs (Rumelhart et al., 1986) are configured in layers, with multiple neuron-like nodes in each layer as illustrated in Figure 1A. Each node gets input from a previous layer weighted by the strengths of synaptic connection and performs a nonlinear transformation of this input. To make the link with predictive coding more visible, we change the direction in which layers are numbered and index the output layer by 0 and the input layer by *l*_max_. We denote by $y_{i}^{\left(l\right)}$ the input to the *i*th node in the *l*th layer, while the transformation of this by an activation function is the output, $f(y_{i}^{\left(l\right)}).$ Thus:

(2.1) $$y_{i}^{\left(l\right)}=\sum_{j=1}^{n^{\left(l+1\right)}}w_{i,j}^{\left(l+1\right)}f\left(y_{j}^{\left(l+1\right)}\right)$$

where $w_{i,j}^{\left(l\right)}$ is the weight from the *j*th node in the *l*th layer to the *i*th node in the (*l* − 1)th layer, and *n*^(*l*)^ is the number of nodes in layer *l*. For brevity, we refer to variable $y_{i}^{\left(l\right)}$ as the activity of a node.

The output the network produces for a given input depends on the values of the weight parameters. This can be illustrated in an example of an ANN shown in Figure 1B. The output node $y_{1}^{\left(0\right)}$ has a high activity as it receives an input from the active input node $y_{1}^{\left(2\right)}$ via strong connections. By contrast, for the other output node $y_{2}^{\left(0\right)},$ there is no path leading to it from the active input node via strong connections, so its activity is low.

The weight values are found during the following training procedure. At the start of each iteration, the activities in the input layer $y_{i}^{\left(l_{\text{max}}\right)}$ are set to values from input training sample, which we denote by $s_{i}^{\mathrm{in}}.$ The network first makes a prediction: the activities of nodes are updated layer by layer according to equation 2.1. The predicted output in the last layer $y_{i}^{\left(0\right)}$ is then compared to the output training sample $s_{i}^{\mathrm{out}}.$ We wish to minimize the difference between the actual and desired output, so we define the following objective function:^1^

(2.2) $$E=-\frac{1}{2}\sum_{i=1}^{n^{\left(0\right)}}{\left(s_{i}^{out}-y_{i}^{\left(0\right)}\right)}^{2}.$$

The training set contains many pairs of training vectors $({\overset{¯}{s}}^{in},{\overset{¯}{s}}^{out})$, which are iteratively presented to the network, but for simplicity of notation, we consider just changes in weights after the presentation of a single training pair. We wish to modify the weights to maximize the objective function, so we update the weights proportionally to the gradient of the objective function,

(2.3) $$\Delta w_{b,c}^{\left(a\right)}=\alpha \frac{\partial E}{\partial w_{b,c}^{\left(a\right)}},$$

where *α* is a parameter describing the learning rate.

Since weight $w_{b,c}^{\left(a\right)}$ determines activity $y_{b}^{\left(a-1\right)},$ the derivative of the objective function over the weight can be found by applying the chain rule:

(2.4) $$\frac{\partial E}{\partial w_{b,c}^{\left(a\right)}}=\frac{\partial E}{\partial y_{b}^{\left(a-1\right)}}\frac{\partial y_{b}^{\left(a-1\right)}}{\partial w_{b,c}^{\left(a\right)}}.$$

The first partial derivative on the right-hand side of equation 2.4 expresses by how much the objective function can be increased by increasing the activity of node *b* in layer *a* − 1, which we denote by

(2.5) $$\delta_{b}^{\left(a-1\right)}=\frac{\partial E}{\partial y_{b}^{\left(a-1\right)}}.$$

The values of these error terms for the sample network in Figure 1B are indicated by the darkness of the arrows labeled $\delta_{i}^{\left(l\right)}.$ The error term $\delta_{2}^{\left(0\right)}$ is high because there is a mismatch between the actual and desired network output, so by increasing the activity in the corresponding node $y_{2}^{\left(0\right)},$ the objective function can be increased. By contrast, the error term $\delta_{1}^{\left(0\right)}$ is low because the corresponding node $y_{1}^{\left(0\right)}$ already produces the desired output, so changing its activity will not increase the objective function. The error term $\delta_{2}^{\left(1\right)}$ is high because the corresponding node $y_{2}^{\left(1\right)}$ projects strongly to the node $y_{2}^{\left(0\right)}$ producing output that is too low, so increasing the value of $y_{2}^{\left(1\right)}$ can increase the objective function. For analogous reasons, the error term $\delta_{1}^{\left(1\right)}$ is low.

Now let us calculate the error terms $\delta_{b}^{\left(a-1\right)}.$ It is straightforward to evaluate them for the output layer:

(2.6) $$\frac{\partial E}{\partial y_{b}^{\left(0\right)}}=s_{b}^{out}-y_{b}^{\left(0\right)}.$$

If we consider a node in an inner layer of the network, then we must consider all possible routes through which the objective function is modified when the activity of the node changes, that is, we must consider the total derivative:

(2.7) $$\frac{\partial E}{\partial y_{b}^{\left(a-1\right)}}=\sum_{i=1}^{n^{\left(a-2\right)}}\frac{\partial E}{\partial y_{i}^{\left(a-2\right)}}\frac{\partial y_{i}^{\left(a-2\right)}}{\partial y_{b}^{\left(a-1\right)}}.$$

Using the definition of equation 2.5 and evaluating the last derivative of equation 2.7 using the chain rule, we obtain the recursive formula for the error terms:

(2.8) $$\delta_{b}^{\left(a-1\right)}=\{\begin{array}{ll} s_{b}^{out}-y_{b}^{\left(a-1\right)} & \text{if}\;a-1=0\\ \sum_{i=1}^{n^{\left(a-2\right)}}\delta_{i}^{\left(a-2\right)}w_{i,b}^{\left(a-1\right)}f\prime \left(y_{b}^{\left(a-1\right)}\right) & \text{if}\;a-1>0 \end{array}.$$

The fact that the error terms in layer *l* > 0 can be computed on the basis of the error terms in the next layer *l* − 1 gave the name “error backpropagation” to the algorithm.

Substituting the definition of error terms from equation 2.5 into equation 2.4 and evaluating the second partial derivative on the right-hand side of equation 2.4, we obtain

(2.9) $$\frac{\partial E}{\partial w_{b,c}^{\left(a\right)}}=\delta_{b}^{\left(a-1\right)}f\left(y_{c}^{\left(a\right)}\right).$$

According to equation 2.9, the change in weight $w_{b,c}^{\left(a\right)}$ is proportional to the product of the output from the presynaptic node $f\left(y_{c}^{\left(a\right)}\right)$ and the error term $\delta_{b}^{\left(a-1\right)}$ associated with the postsynaptic node. Red upward-pointing arrows in Figure 1B indicate which weights would be increased the most in this example, and it is evident that the increase in these weights will indeed increase the objective function.

In summary, after presenting to the network a training sample, each weight is modified proportionally to the gradient given in equation 2.9 with the error term given by equation 2.8. The expression for weight change (see equations 2.9 and 2.8) is a complex global function of activities and weights of neurons not connected to the synapse being modified. In order for real neurons to compute it, the architecture of the model could be extended to include nodes computing the error terms, which could affect the weight changes. As we will see, analogous nodes are present in the predictive coding model.

### 2.2 Predictive Coding for Supervised Learning

Due to the generality of the predictive coding framework, multiple network architectures within this framework can perform supervised learning. In this section, we describe the simplest model that can closely approximate the backpropagation; we consider other architectures later. The description in this section closely follows that of unsupervised predictive coding networks (Rao & Ballard, 1999; Friston, 2005) but is adapted for the supervised setting. Also, we provide a succinct description of the model. For readers interested in a gradual and more detailed introduction to the predictive coding framework, we recommend reading sections 1 and 2 of a tutorial on this framework (Bogacz, 2017) before reading this section.

We first propose a probabilistic model for supervised learning. Then we describe the inference in the model, its neural implementation, and finally learning of model parameters.

#### 2.2.1 Probabilistic Model

**Figure 2:**
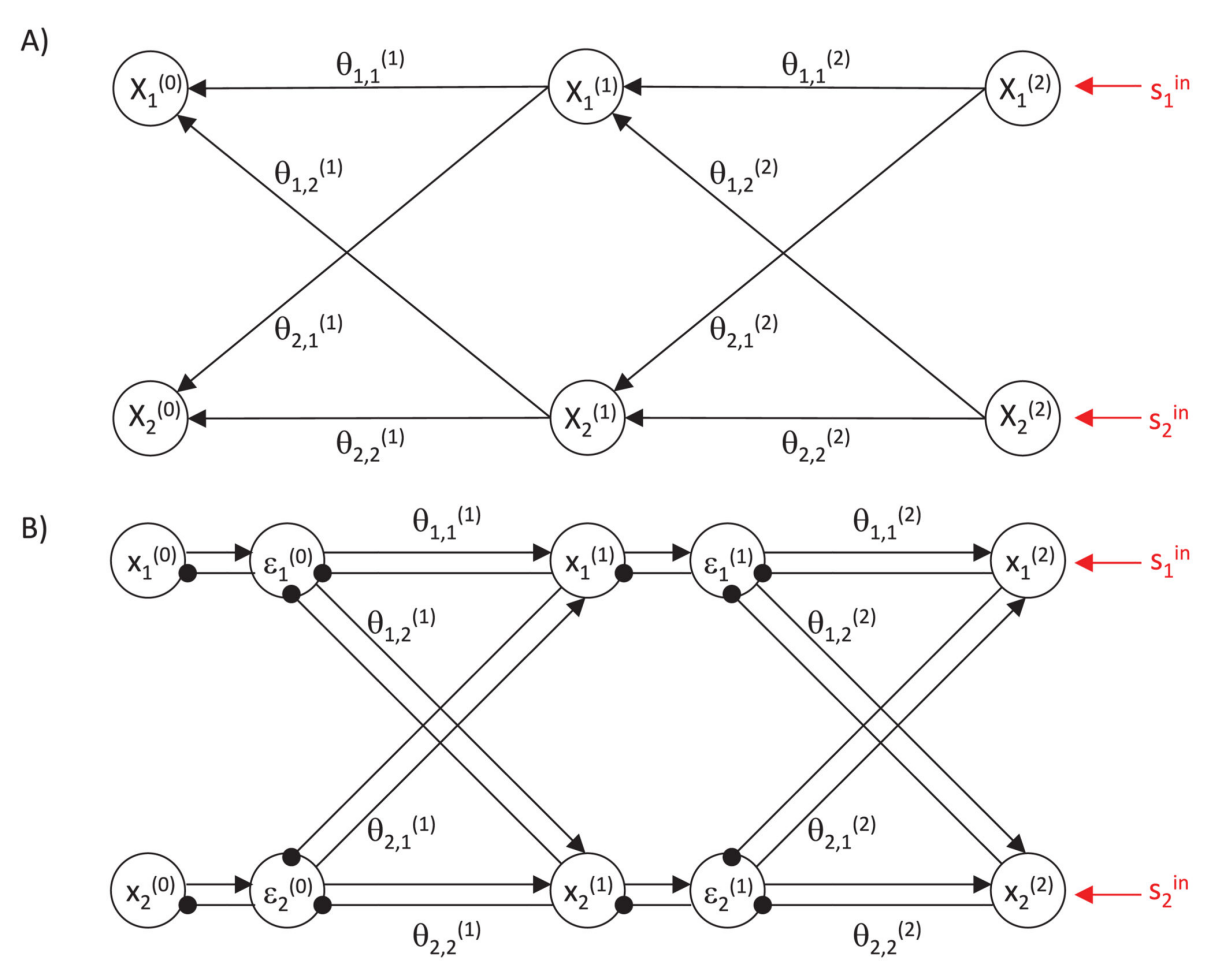
Predictive coding model. (A) Structure of the probabilistic model. Circles denote random variables, and arrows denote dependencies between them. (B) Architecture of the network. Arrows and lines ending with circles denote excitatory and inhibitory connections, respectively. Connections without labels have weights fixed to 1.

Figure 2A shows a structure of a probabilistic model that parallels the architecture of the ANN shown in Figure 1A. It consists of *l*_max_ layers of variables, such that the variables on level *l* depend on the variables on level *l* + 1. It is important to emphasize that Figure 2A does not show the architecture of the predictive coding network, only the structure of the underlying probabilistic model. As we will see, the inference in this model can be implemented by a network with the architecture shown in Figure 2B.

By analogy to ANNs, we assume that variables on the highest level $X_{i}^{\left(l_{\text{max}}\right)}$ are fixed to the input sample $s_{i}^{in},$ and the inferred values of variables on level 0 are the output from the network. Readers familiar with predictive coding models for sensory processing may be surprised that the sensory input is provided to the highest level; traditionally in these models, the input is provided to level 0. Indeed, when biological neural networks learn in a supervised manner, both input and output are provided to sensory cortices. For example, when a child learns the sounds of the letters, the input (i.e., the shape of a letter) is provided to visual cortex, the output (i.e., the sound) is provided to the auditory cortex, and both of these sensory cortices communicate with associative cortex. The model we consider in this section corresponds to a part of this network: from associative areas to the sensory modality to which the output is provided. So in the example, level 0 corresponds to the auditory cortex, while the highest levels correspond to associative areas. Thus, the input $s_{i}^{in}$ presented to this network corresponds not to the raw sensory input but, rather, to its representation preprocessed by visual networks. We discuss how the sensory networks processing the input modality can be introduced to the model in section 3.

Let ${\overset{¯}{X}}^{\left(l\right)}$ be a vector of random variables on level *l*, and let us denote a sample from random variable ${\overset{¯}{X}}^{\left(l\right)}$ by ${\overset{¯}{x}}^{\left(l\right)}$. Let us assume the following relationship between the random variables on adjacent levels (for brevity of notation, we write $P({\overset{¯}{x}}^{\left(l\right)})$ instead of $P({\overset{¯}{X}}^{\left(l\right)}={\overset{¯}{x}}^{\left(l\right)})$):

(2.10) $$P\left(x_{i}^{\left(l\right)}|{\bar{x}}^{\left(l+1\right)}\right)=𝒩\left(x_{i}^{\left(l\right)};\mu_{i}^{\left(l\right)},\Sigma_{i}^{\left(l\right)}\right).$$

In equation 2.10, 𝒩 (*x*; *μ*, Σ) is the probability density of a normal distribution with mean *μ* and variance Σ. The mean of probability density on level *l* is a function of the values on the higher-level analogous to the input to a node in ANN (see equation 2.1):

(2.11) $$\mu_{i}^{\left(l\right)}=\sum_{j=1}^{n^{\left(l+1\right)}}\theta_{i,j}^{\left(l+1\right)}f\left(x_{j}^{\left(l+1\right)}\right).$$

In equation 2.11, *n*^(*l*)^ denotes the number of random variables on level *l*, and $\theta_{i,j}^{\left(l+1\right)}$ are the parameters describing the dependence of random variables. For simplicity in this letter, we do not consider how $\Sigma_{i}^{\left(l\right)}$ are learned (Friston, 2005; Bogacz, 2017), but treat them as fixed parameters.

#### 2.2.2 Inference

We now move to describing the inference in the model: finding the most likely values of model variables, which will determine the activity of nodes in the predictive coding network. We wish to find the most likely values of all unconstrained random variables in the model that maximize the probability $P\left({\bar{x}}^{\left(0\right)},\ldots ,{\bar{x}}^{\left(l_{\text{max}}-1\right)}|{\bar{x}}^{\left(l_{\text{max}}\right)}\right)$ (see Friston, 2005, and Bogacz, 2017, for the technical details, however we are only considering the first moment of an approximate distribution for each random variable and from now onwards we will use the same notation $x_{i}^{\left(l\right)}$ to describe the first moments). Since the nodes on the highest levels are fixed to $x_{i}^{\left(l_{\text{max}}\right)}=s_{i}^{in},$ their values are not being changed but, rather, provide a condition on other variables. To simplify calculations, we define the objective function equal to the logarithm of the joint distribution (since the logarithm is a monotonic operator, a logarithm of a function has the same maximum as the function itself):

(2.12) $$F=\text{ln}\left(P\left({\bar{x}}^{\left(0\right)},\ldots \;,{\bar{x}}^{\left(l_{\text{max}}-1\right)}|{\bar{x}}^{\left(l_{\text{max}}\right)}\right)\right).$$

Since we assumed that the variables on one level depend on variables of the level above, we can write the objective function as

(2.13) $$F=\sum_{l=0}^{l_{\text{max}}-1}\text{ln}\left(P\left({\bar{x}}^{\left(l\right)}|{\bar{x}}^{\left(l+1\right)}\right)\right).$$

Substituting equation 2.10 and the expression for a normal distribution into the above equation, we obtain:

(2.14) $$F=\sum_{l=0}^{l_{\text{max}}-1}\sum_{i=1}^{n^{\left(l\right)}}\left[\text{ln}\frac{1}{\sqrt{2\pi }\Sigma_{i}^{\left(l\right)}}-\frac{{\left(x_{i}^{\left(l\right)}-\mu_{i}^{\left(l\right)}\right)}^{2}}{2\Sigma_{i}^{\left(l\right)}}\right].$$

Then, ignoring constant terms, we can write the objective function as

(2.15) $$F=-\frac{1}{2}\sum_{l=0}^{l_{\text{max}}-1}\sum_{i=1}^{n^{\left(l\right)}}\frac{{\left(x_{i}^{\left(l\right)}-\mu_{i}^{\left(l\right)}\right)}^{2}}{\Sigma_{i}^{\left(l\right)}}.$$

Recall that we wish to find the values $x_{i}^{\left(l\right)}$ that maximize the above objective function. This can be achieved by modifying $x_{i}^{\left(l\right)}$ proportionally to the gradient of the objective function. To calculate the derivative of *F* over $x_{i}^{\left(l\right)}$ we note that each $x_{i}^{\left(l\right)}$ influences *F* in two ways: it occurs in equation 2.15 explicitly, but it also determines the values of $\mu_{j}^{\left(l-1\right)}.$ Thus, the derivative contains two terms:

(2.16) $$\frac{\partial F}{\partial x_{b}^{\left(a\right)}}=-\frac{x_{b}^{\left(a\right)}-\mu_{b}^{\left(a\right)}}{\Sigma_{b}^{\left(a\right)}}+\sum_{i=1}^{n^{\left(a-1\right)}}\frac{x_{i}^{\left(a-1\right)}-\mu_{i}^{\left(a-1\right)}}{\Sigma_{i}^{\left(a-1\right)}}\theta_{i,b}^{\left(a\right)}f^{\prime }\left(x_{b}^{\left(a\right)}\right).$$

In equation 2.16, there are terms that repeat, so we denote them by

(2.17) $$\varepsilon_{i}^{\left(l\right)}=\frac{x_{i}^{\left(l\right)}-\mu_{i}^{\left(l\right)}}{\Sigma_{i}^{\left(l\right)}}.$$

These terms describe by how much the value of a random variable on a given level differs from the mean predicted by a higher level, so we refer to them as prediction errors. Substituting the definition of prediction errors into equation 2.16, we obtain the following rule describing changes in $x_{b}^{\left(a\right)}$ over time:

(2.18) $${\dot{x}}_{b}^{\left(a\right)}=-\varepsilon_{b}^{\left(a\right)}+\sum_{i=1}^{n^{\left(a-1\right)}}\varepsilon_{i}^{\left(a-1\right)}\theta_{i,b}^{\left(a\right)}f^{\prime }\left(x_{b}^{\left(a\right)}\right).$$

#### 2.2.3 Neural Implementation

The computations described by equations 2.17 and 2.18 could be performed by a simple network illustrated in Figure 2B with nodes corresponding to prediction errors $\varepsilon_{i}^{\left(l\right)}$ and values of random variables $x_{i}^{\left(l\right)}.$ The prediction errors $\varepsilon_{i}^{\left(l\right)}$ are computed on the basis of excitation from corresponding variable nodes $x_{i}^{\left(l\right)}$ and inhibition from the nodes on the higher level $x_{j}^{\left(l+1\right)}$ weighted by strength of synaptic connections $\theta_{i,j}^{\left(l+1\right)}.$ Conversely, the nodes $x_{i}^{\left(l\right)}$ make computations on the basis of the prediction error from the corresponding level and the prediction errors from the lower level weighted by synaptic weights.

It is important to emphasize that for a linear function *f* (*x*) = *x*, the nonlinear terms in equations 2.17 and 2.18 would disappear, and these equations could be fully implemented in the simple network shown in Figure 2B. To implement equation 2.17, a prediction error node would get excitation from the corresponding variable node and inhibition equal to synaptic input from higher-level nodes; thus, it could compute the difference between them. Scaling the activity of nodes encoding prediction error by a constant $\Sigma_{i}^{\left(l\right)}$ could be implemented by self-inhibitory connections with weight $\Sigma_{i}^{\left(l\right)}$ (we do not consider them here for simplicity: for details see Friston, 2005, and Bogacz, 2017). Analogous to implementing equation 2.18, a variable node would need to change its activity proportionally to its inputs.

**Figure 3:**
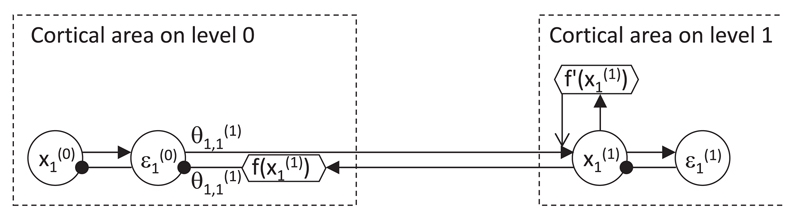
Possible implementation of nonlinearities in the predictive coding model (magnification of a part of the network in Figure 2B). Filled arrows and lines ending with circles denote excitatory and inhibitory connections, respectively. Open arrow denotes a modulatory connection with multiplicative effect. Circles and hexagons denote nodes performing linear and nonlinear computations, respectively.

One can imagine several ways that the nonlinear terms can be implemented, and Figure 3 shows one of them (Bogacz, 2017). The prediction error nodes need to receive the input from the higher-level nodes transformed through a nonlinear function, and this transformation could be implemented by additional nodes (indicated by a hexagon labeled $f(x_{1}^{\left(1\right)})$ in Figure 3). Introducing additional nodes is also necessary to make the pattern of connectivity in the model more consistent with that observed in the cortex. In particular, in the original predictive coding architecture (see Figure 2B), the projections from the higher levels are inhibitory, whereas connections between cortical areas are excitatory. Thus, to make the predictive coding network in accordance with this, the sign of the top-down input needs to be inverted by local inhibitory neurons (Spratling, 2008). Here we propose that these local inhibitory neurons could additionally perform a nonlinear transformation. With this arrangement, there are individual nodes encoding $x_{b}^{\left(a\right)}$ and $f(x_{b}^{\left(a\right)}),$ and each node sends only the value it encodes. According to equation 2.18, the input from the lower level to a variable node needs to be scaled by a nonlinear function of the activity of variable node itself. Such scaling could be implemented by either a separate node (indicated by a hexagon labeled $f^{\prime }(x_{1}^{\left(1\right)})$ in Figure 3) or intrinsic mechanisms within the variable node that would make it react to excitatory inputs differentially depending on its own activity level.

In the predictive coding model, after the input is provided, all nodes are updated according to equations 2.17 and 2.18, until the network converges to a steady state. We label variables in the steady state with an asterisk (e.g., $x_{i}^{*(l)}$ or F*).

**Figure 4:**
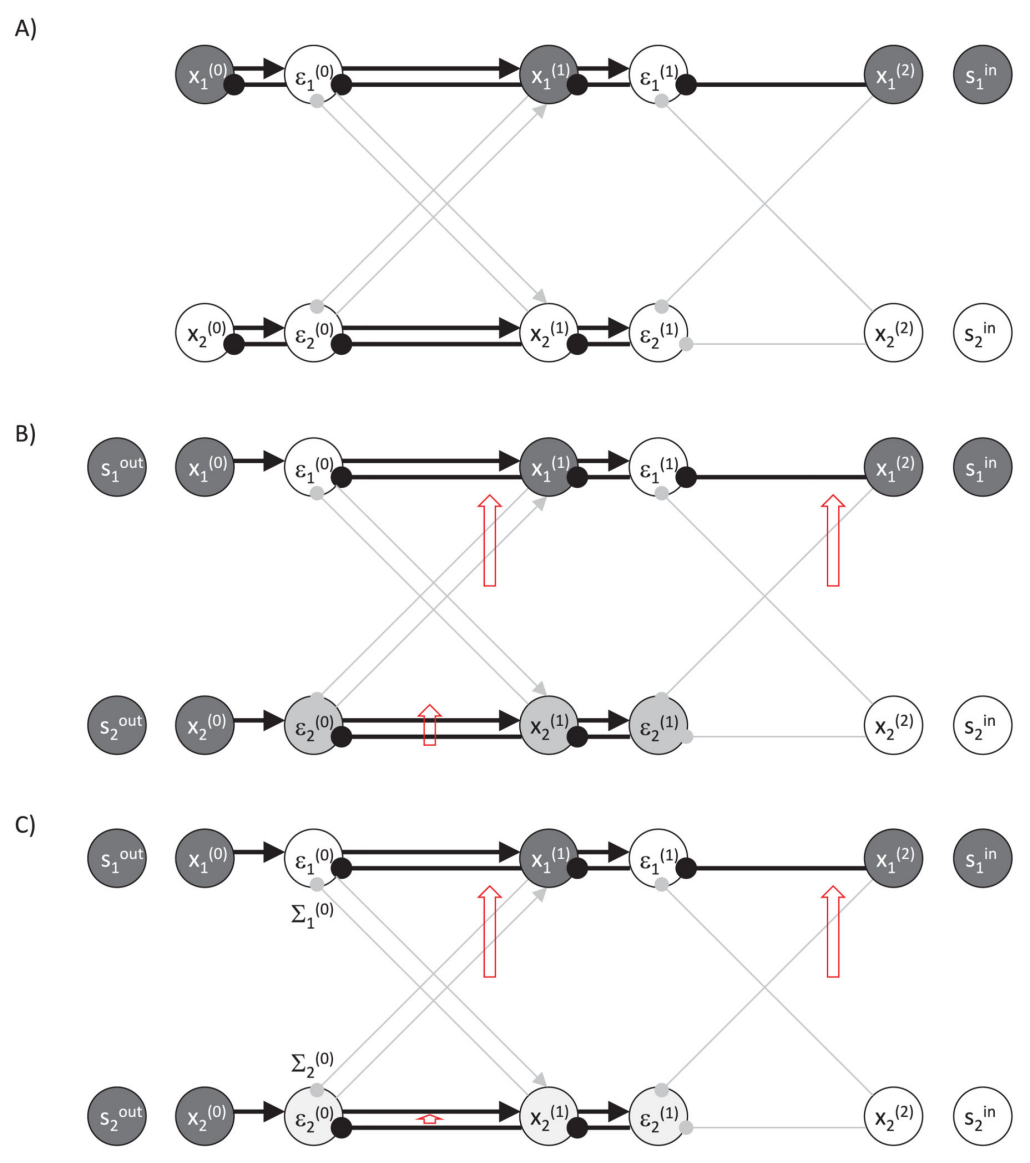
Example of a predictive coding network for supervised learning. (A) Prediction mode. (B) Learning mode. (C) Learning mode for a network with high value of parameter describing sensory noise. Notation as in Figure 2B.

Figure 4A illustrates values to which a sample model converges when presented with a sample pattern. The activity in this case propagates from $x_{1}^{\left(2\right)}$ node through the connections with high weights, resulting in activation of nodes $x_{1}^{\left(1\right)}$ and $x_{1}^{\left(0\right)}$ (note that the double inhibitory connection from higher to lower levels has overall excitatory effect). Initially the prediction error nodes would change their activity, but eventually their activity converges to 0 as their excitatory input becomes exactly balanced by inhibition.

#### 2.2.4 Learning Parameters

During learning, the values of the nodes on the lowest level are set to the output sample, ${\overset{¯}{x}}^{\left(0\right)}={\overset{¯}{s}}^{out}$ , as illustrated in Figure 4B. Then the values of all nodes on levels *l* ∈ {1,…, *l*_max_ − 1} are modified in the same way as described before (see equation 2.18).

Figure 4B illustrates an example of operation of the model. The model is presented with the desired output in which both nodes $x_{1}^{\left(0\right)}$ and $x_{2}^{\left(0\right)}$ are active. Node $x_{1}^{\left(1\right)}$ becomes active as it receives both top-down and bottom-up input. There is no mismatch between these inputs, so the corresponding prediction error nodes ($\varepsilon_{1}^{\left(0\right)}$ and $\varepsilon_{1}^{\left(1\right)}$) are not active. By contrast, the node $x_{2}^{\left(1\right)}$ gets bottom-up but no top-down input, so its activity has intermediate value, and the prediction error nodes connected with it ($\varepsilon_{2}^{\left(0\right)}$ and $\varepsilon_{2}^{\left(1\right)}$) are active.

Once the network has reached its steady state, the parameters of the model $\theta_{i,j}^{\left(l\right)}$ are updated, so the model better predicts the desired output. This is achieved by modifying $\theta_{i,j}^{\left(l\right)}$ proportionally to the gradient of the objective function over the parameters. To compute the derivative of the objective function over $\theta_{i,j}^{\left(l\right)},$ we note that $\theta_{i,j}^{\left(l\right)}$ affects the value of function *F* of equation 2.15 by influencing $\mu_{i}^{\left(l-1\right)},$ hence

(2.19) $$\frac{\partial F*}{\partial \theta_{b,c}^{\left(a\right)}}=\varepsilon_{b}^{*(a-1)}f\left(x_{c}^{*(a)}\right).$$

According to equation 2.19, the change in a synaptic weight $\theta_{b,c}^{\left(a\right)}$ of connection between levels *a* and *a* − 1 is proportional to the product of quantities encoded on these levels. For a linear function *f* (*x*) = *x*, the nonlinearity

**Algorithm 1: Pseudocode for Predictive Coding During Learning.**

**for all** Data **do**

     ${\overset{¯}{x}}^{(0)}\;\leftarrow \;{\overset{¯}{s}}^{out}$

     ${\overset{¯}{x}}^{(l_{\text{max}})}\;\leftarrow \;{\overset{¯}{s}}^{in}$

     **repeat**

          Inference: Equations 2.17, 2.18

     **until** convergence

     Update weights: Equation 2.19

In the simulations presented, to make for faster simulation, first a prediction was made by inputting *s̄*^*in*^ alone and propagating through the network layer by layer, as we know that all error nodes eventually would converge to zero in the prediction phase (see section 3). Then the output *s̄^out^* is applied, after which inference took place.

in equation 2.19 would disappear, and the weight change would simply be equal to the product of the activities of presynaptic and postsynaptic nodes (see Figure 2B). Even if the nonlinearity is considered, as in Figure 3, the weight change is fully determined by the activity of presynaptic and postsynaptic nodes. The learning rules of the top and bottom weights must be slightly different. For the bottom connection labeled $\theta_{1,1}^{\left(1\right)}$ in Figure 3, the change in a synaptic weight is simply equal to the product of the activity of nodes it connects (round node $\varepsilon_{1}^{\left(0\right)}$ and hexagonal node $f(x_{1}^{\left(1\right)})).$ For the top connection, the change in weights is equal to the product of activity of the presynaptic node $(\varepsilon_{1}^{\left(0\right)})$ and function *f* of activity of the postsynaptic node (round node $x_{1}^{\left(1\right)}$). This then maintains the symmetry of the connections: the bottom and the top connections are modified by the same amount. We refer to these changes as Hebbian in a sense that in both cases, the weight change is a product of monotonically increasing functions of activity of presynaptic and postsynaptic neurons.

Figure 4B illustrates the resulting changes in the weights. In the example in Figure 4B, the weights that increase the most are indicated by long red upward arrows. There would also be an increase in the weight between $\varepsilon_{2}^{\left(0\right)}$ and $x_{2}^{\left(1\right)},$ indicated by a shorter arrow, but it would be not as large as node $x_{2}^{\left(1\right)}$ has lower activity. It is evident that after these weight changes, the activity of prediction error nodes would be reduced indicating that the desired output is better predicted by the network. In algorithm 1, we include pseudocode to clarify how the network operates in training mode.

## 3 Results

### 3.1 Relationship between the Models

An ANN has two modes of operation: during prediction, it computes its output on the basis of${\overset{¯}{s}}^{in}$, while during learning, it updates its weights on the basis of ${\overset{¯}{s}}^{in}$ and ${\overset{¯}{s}}^{out}$. The predictive coding network can also operate in these modes. We next discuss the relationship between computations of an ANN and a predictive coding network in these two modes.

#### 3.1.1 Prediction

We show that the predictive coding network has a stable fixed point at the state where all nodes have the same values as the corresponding nodes in the ANN receiving the same input ${\overset{¯}{s}}^{in}$. Since all nodes change proportionally to the gradient of *F*, the value of function *F* always increases. Since the network is constrained only by the input, the maximum value that *F* can reach is 0; because *F* is a negative of sum of squares, this maximum is achieved if all terms in the summation of equation 2.15 are equal to 0, that is, when

(3.1) $$x_{i}^{*(l)}=\mu_{i}^{*(l)}$$

Since $\mu_{i}^{\left(l\right)}$ is defined in analogous way as $y_{i}^{\left(l\right)}$ (cf. equations 2.1 and 2.11), the nodes in the prediction mode have the same values at the fixed point as the corresponding nodes in the ANN: $x_{i}^{*(l)}=y_{i}^{\left(l\right)}$.

The above property is illustrated in Figure 4A, in which weights are set to the same value as for the ANN in Figure 1B, and the network is presented with the same input sample. The network converges to the same pattern of activity on level *l* = 0 as for the ANN in Figure 1B.

#### 3.1.2 Learning

The pattern of weight change in the predictive coding network shown in Figure 4B is similar as in the backpropagation algorithm (see Figure 1B). We now analyze under what conditions weight changes in the predictive coding model converge to that in the backpropagation algorithm.

The weight update rules in the two models (see equations 2.9 and 2.19) have the same form; however, the prediction error terms $\delta_{i}^{\left(l\right)}$ and $\varepsilon_{i}^{\left(l\right)}$ were defined differently. To see the relationship between these terms, we now derive the recursive formula for prediction errors $\varepsilon_{i}^{\left(l\right)}$ analogous to that for $\delta_{i}^{\left(l\right)}$ in equation 2.8. We note that once the network reaches the steady state in the learning mode, the change in activity of each node must be equal to zero. Setting the left-hand side of equation 2.18 to 0, we obtain

(3.2) $$\varepsilon_{b}^{*(a)}=\sum_{i=1}^{n^{\left(a-1\right)}}\varepsilon_{i}^{*(a-1)}\theta_{i,b}^{\left(a\right)}f^{\prime }\left(x_{b}^{*(a)}\right).$$

We can now write a recursive formula for the prediction errors:

(3.3) $$\varepsilon_{b}^{*(a-1)}=\{\begin{array}{ll} \left(s_{b}^{out}-\mu_{b}^{*(a-1)}\right)/\Sigma_{b}^{\left(0\right)} & \text{if}\;a-1=0\\ \sum_{i=1}^{n^{\left(a-2\right)}}\varepsilon_{i}^{*(a-2)}\theta_{i,b}^{\left(a-1\right)}f^{\prime }\left(x_{b}^{*(a-1)}\right) & \text{if}\;a-1>0 \end{array}.$$

We first consider the case when all variance parameters are set to $\Sigma_{i}^{\left(l\right)}=1$ (this corresponds to the original model of Rao & Ballard, 1999, where the prediction errors were not normalized). Then the formula has exactly the same form as for the backpropagation algorithm, equation 2.8. Therefore, it may seem that the weight change in the two models is identical. However, for the weight change to be identical, the values of the corresponding nodes must be equal: $x_{i}^{*(l)}=y_{i}^{\left(l\right)}$ (it is sufficient for this condition to hold for *l* > 0, because $x_{i}^{*(0)}$ do not directly influence weight changes). Although we have shown in that $x_{i}^{*(l)}=y_{i}^{\left(l\right)}$ in the prediction mode, it may not be the case in the learning mode, because the nodes $x_{i}^{\left(0\right)}$ are fixed (to $s_{i}^{out}$), and thus function *F* may not reach the maximum of 0, so equation 3.1 may not be satisfied.

We now consider under what conditions $x_{i}^{*(l)}$ is equal or close to $y_{i}^{\left(l\right)}.$ First, when the networks are trained such that they correctly predict the output training samples, then objective function *F* can reach 0 during the relaxation and hence $x_{i}^{*(l)}=y_{i}^{\left(l\right)},$ and the two models have exactly the same weight changes. In particular, the change in weights is then equal to 0; thus, the weights resulting in perfect prediction are a fixed point for both models.

Second, when the networks are trained such that their predictions are close to the output training samples, then fixing $x_{i}^{\left(0\right)}$ will only slightly change the activity of other nodes in the predictive coding model, so the weight change will be similar.

**Figure 5:**
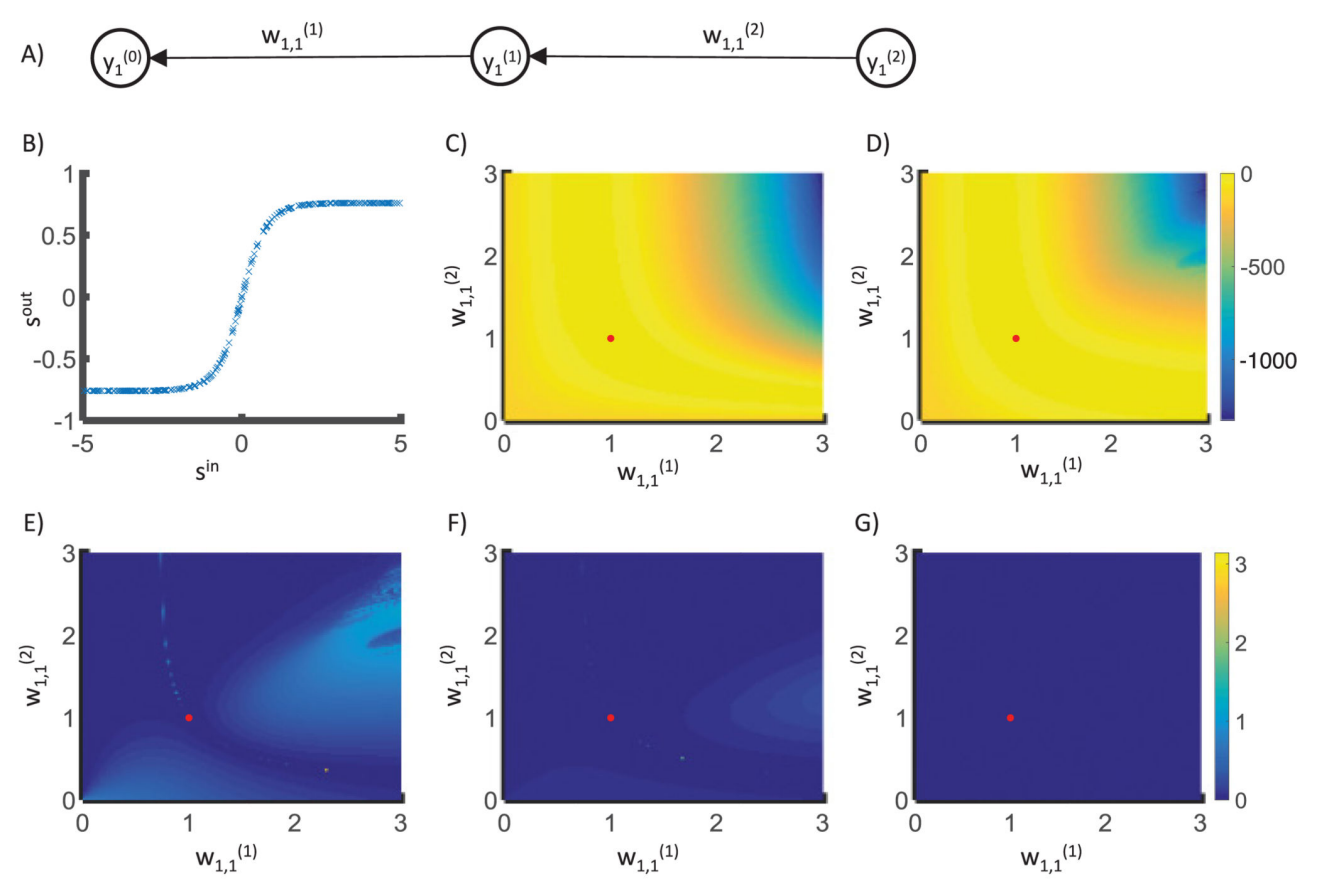
Comparison of weight changes in backpropagation and predictive coding models. (A) The structure of the network used. (B) The data that the models were trained on—here, *s^out^* = tanh(tanh(*s^in^*)). (C) The objective function of an ANN for a training set with 300 samples generated as described. The objective function is equal to the sum of 300 terms given by equation 2.2 corresponding to individual training samples. The red dot indicates weights that maximize the objective function. (D) The objective function of the predictive coding model at the fixed point. For each set of weights and training sample, to find the state of predictive coding network at the fixed point, the nodes in layers 0 and 2 were set to training examples, and the node in layer 1 was updated according to equation 2.18. This equation was solved using the Euler method. A dynamic form of the Euler integration step was used where its size was allowed to reduce by a factor should the system not be converging (i.e., the maximum change in node activity increases from the previous step). The initial step size was 0.2. The relaxation was performed until the maximum value of $\partial F/\partial x_{i}^{\left(l\right)}$ was lower than $10^{-6}/\Sigma_{i}^{\left(0\right)}$ or 1 million iterations had been performed. (E–G) Angle difference between the gradient for the ANN and the gradient for the predictive coding model found from equation 2.19. Different panels correspond to different values of parameter describing sensory noise. (E) $\Sigma_{1}^{\left(0\right)}=1.$ (F) $\Sigma_{1}^{\left(0\right)}=8.$ (G) $\Sigma_{1}^{\left(0\right)}=256.$

To illustrate this property, we compare the weight changes in predictive coding models and ANN with the very simple architecture shown in Figure 5A. This network consists of just three layers (*l*_max_ = 2) and one node in each layer (*n*^(0)^ = *n*^(1)^ = *n*^(2)^ = 1). Such a network has only two weight parameters ($w_{1,1}^{\left(1\right)}$ and $w_{1,1}^{\left(2\right)}$), so the objective function of the ANN can be easily visualized. The network was trained on a set in which input training samples were generated randomly from uniform distribution $s_{1}^{in}$ ∈ [−5, 5], and output training samples were generated as $s_{1}^{out}=W^{\left(1\right)}\;\tanh(W^{\left(2\right)}\;\tanh(s_{i}^{in})),$ where *W*^(1)^ = *W*^(2)^ = 1 (see Figure 5B). Figure 5C shows the objective function of the ANN for this training set. Thus, an ANN with weights equal to $w_{1,1}^{\left(l\right)}=W^{\left(l\right)}$ perfectly predicts all samples in the training set, so the objective function is equal to 0. There are also other combinations of weights resulting in good prediction, which create a ridge of the objective function.

Figure 5E shows the angle between the direction of weight change in backpropagation and the predictive coding model. The directions of the gradient for the two models are very similar except for the regions where the objective functions *E* and F* are misaligned (see Figures 5C and 5D). Nevertheless, close to the maximum of the objective function (indicated by a red dot), the directions of weight change become similar and the angle decreases toward 0.

There is also a third condition under which the predictive coding network approximates the backpropagation algorithm. When the value of parameters $\Sigma_{i}^{\left(0\right)}$ is increased relative to other $\Sigma_{i}^{\left(l\right)},$ the impact of fixing $x_{i}^{\left(0\right)}$ on the activity of other nodes is reduced, because $\varepsilon_{i}^{\left(0\right)}$ becomes smaller (see equation 2.17) and its influence on activity of other nodes is reduced. Thus $x_{i}^{*(l)}$ is closer to $y_{i}^{\left(l\right)}.$ (for *l* > 0), and the weight change in the predictive coding model becomes closer to that in the backpropagation algorithm (recall that the weight changes are the same when $x_{i}^{*(l)}=y_{i}^{\left(l\right)}$ for *l* > 0).

Multiplying $\Sigma_{i}^{\left(0\right)}$ by a constant will also reduce all $\varepsilon_{i}^{\left(l\right)}$ by the same constant (see equation 3.3); consequently, all weight changes will be reduced by this constant. This can be compensated by multiplying the learning rate *α* by the same constant, so the magnitude of the weight change remains constant. In this case, the weight updates of the predictive coding network will become asymptotically similar to the ANN, regardless of prediction accuracy.

Figures 5F and 5G show that as $\Sigma_{i}^{\left(0\right)}$ increases, the angle between weight changes in the two models decreases toward 0. Thus, as the parameters $\Sigma_{i}^{\left(0\right)}$ are increased, the weight changes in the predictive coding model converge to those in the backpropagation algorithm.

**Figure 6:**
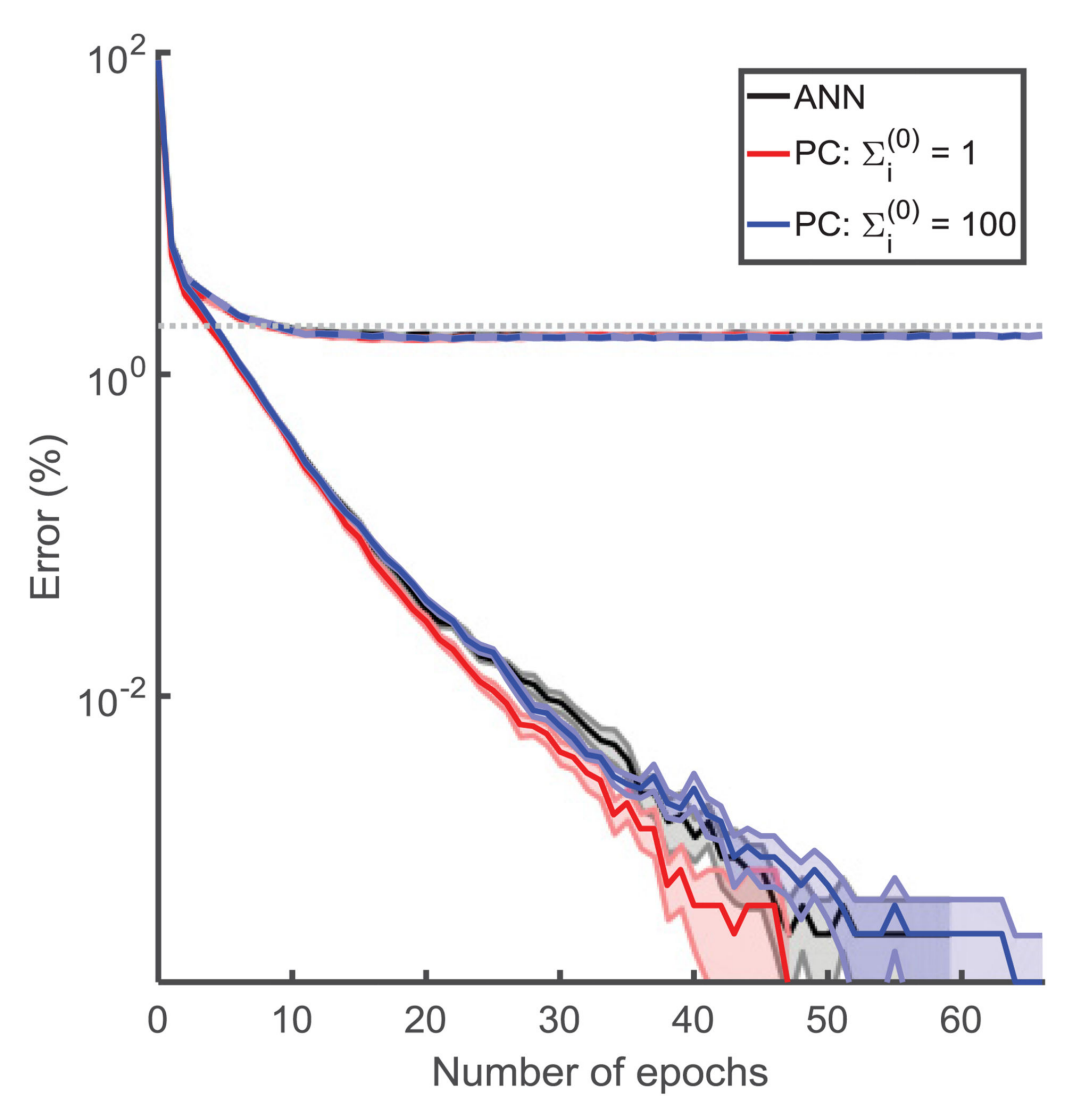
Comparison of prediction accuracy (%) for different models (indicated by colors; see the key) on the MNIST dataset. Training errors are shown with solid lines and validation errors with dashed lines. The dotted gray line denotes 2% error. The models were run 10 times each, initialized with different weights. When the training error lines stop, this is when the mean error of the 10 runs was equal to zero. The weights were drawn from a uniform distribution with maximum and minimum values of $\pm 4\sqrt{\frac{6}{N}},$ where *N* is the total number of neurons in the two layers on either side of the weight. The input data were first transformed through an inverse logistic function as preprocessing before being given to the network. When the network was trained with an image of class *c*, the nodes in layer 0 were set to $x_{c}^{\left(0\right)}=0.97$ and $x_{j\neq c}^{\left(0\right)}=0.03.$ After inference and before the weight update, the error node values were scaled by $\Sigma_{i}^{\left(0\right)}$ so as to be able to compare between the models. We used a batch size of 20, with a learning rate of 0.001 and the stochastic optimizer Adam (Kingma & Ba, 2014) to accelerate learning; this is essentially a per parameter learning rate, where weights that are infrequently updated are updated more and vice versa. We chose the number of steps in the inference phase to be 20; at this point, the network will not necessarily have converged, but we did so to aid speed of training. This is not the minimum number of inference iterations that allows for good learning, a notion that we will explore in a future paper. Otherwise simulations are according to Figure 5. The shaded regions in the fainter color describe the standard error of the mean. The figure is shown on a logarithmic plot.

Figure 4C illustrates the impact of increasing $\Sigma_{i}^{\left(0\right)}.$ It reduces $\varepsilon_{2}^{\left(0\right)},$ which in turn reduces $x_{2}^{\left(1\right)}$ and $\varepsilon_{2}^{\left(1\right)}.$ This decreases all weight changes, particularly the change of the weight between nodes $\varepsilon_{2}^{\left(0\right)}$ and $x_{2}^{\left(1\right)}$ (indicated by a short red arrow) because both of these nodes have reduced activity. After compensating for the learning rate, these weight changes become more similar to those in the backpropagation algorithm (compare Figures 4B, 4C, and 1B). However, we note that learning driven by very small values of the error nodes is less biologically plausible. In Figure 6, we will show that a high value of $\Sigma_{i}^{\left(0\right)}$ is not required for good learning with these networks.

### 3.2 Performance on More Complex Learning Tasks

To efficiently learn in more complex tasks, ANNs include a “bias term,” or an additional node in each layer that does not receive any input but has activity equal to 1. We define this node as the node with index 0 in each layer, so $f(y_{0}^{\left(l\right)})=1.$ With such a node, the definition of synaptic input (see equation 2.1) is extended to include one additional term $w_{i,0}^{\left(l+1\right)},$ which is referred to as the bias term. The weight corresponding to the bias term is updated during learning according to the same rule as all other weights (see equation 2.9).

An equivalent bias term can be easily introduced to the predictive coding models. This would be just a node with a constant output of $f(x_{0}^{\left(l\right)})=1,$ which projects to the next layer but does have an associated error node. The activity of such a node would not change after the training inputs are provided, and corresponding weights $\theta_{i,0}^{\left(l+1\right)}$ would be modified like all other weights (see equation 2.19).

To assess the performance of the predictive coding model on more complex learning tasks, we tested it on the MNIST data set. This is a data set of 28 by 28 images of handwritten digits, each associated with one of the 10 corresponding classes of digits. We performed the analysis for an ANN of size 784-600-600-10 (*l*_max_ = 3), with predictive coding networks of the corresponding size. We use the logistic sigmoid as the activation function. We ran the simulations for both the $\Sigma_{i}^{\left(0\right)}=1$ case and the $\Sigma_{i}^{\left(0\right)}=100$ case. Figure 6 shows the learning curves for these different models. Each curve is the mean from 10 simulations, with the standard error shown as the shaded regions.

We see that the predictive coding models perform similarly to the ANN. For a large value of parameter $\Sigma_{i}^{\left(0\right)},$ the performance of the predictive coding model was very similar to the backpropagation algorithm, in agreement with an earlier analysis showing that the weight changes in the predictive coding model then converge to those in the backpropagation algorithm. Should we have had more than 20 steps in each inference stage (i.e., allowed the network to converge in inference), the ANN and the predictive coding network with $\Sigma_{i}^{\left(0\right)}=100$ would have had an even more similar trajectory.

We see that all the networks eventually obtain a training error of 0.00% and a validation error of 1.7% to 1.8%. We did not optimize the learning rate for validation error as we are solely highlighting the similarity between ANNs and predictive coding.

### 3.3 Effects of the Architecture of the Predictive Coding Model

Since the networks we have considered so far corresponded to the associative areas and sensory area to which the output sample was provided, the input samples $s_{i}^{in}$ were provided to the nodes at the highest level of hierarchy, so we assumed that sensory inputs are already preprocessed by sensory areas. The sensory areas can be added to the model by considering an architecture in which there are two separate lower-level areas receiving $s_{i}^{in}$ and $s_{i}^{out},$ which are both connected with higher areas (de Sa & Ballard, 1998; Hyvarinen, 1999; O’Reilly & Munakata, 2000; Larochelle & Bengio, 2008; Bengio, 2014; Srivastava & Salakhutdinov, 2012; Hinton, Osindero, & Teh, 2006). For example, in case of learning associations between visual stimuli (e.g., shapes of letters) and auditory stimuli (e.g., their sounds), $s_{i}^{in}$ and $s_{i}^{out}$ could be provided to primary visual and primary auditory cortices, respectively. Both of these primary areas project through a hierarchy of sensory areas to a common higher associative cortex.

**Figure 7:**
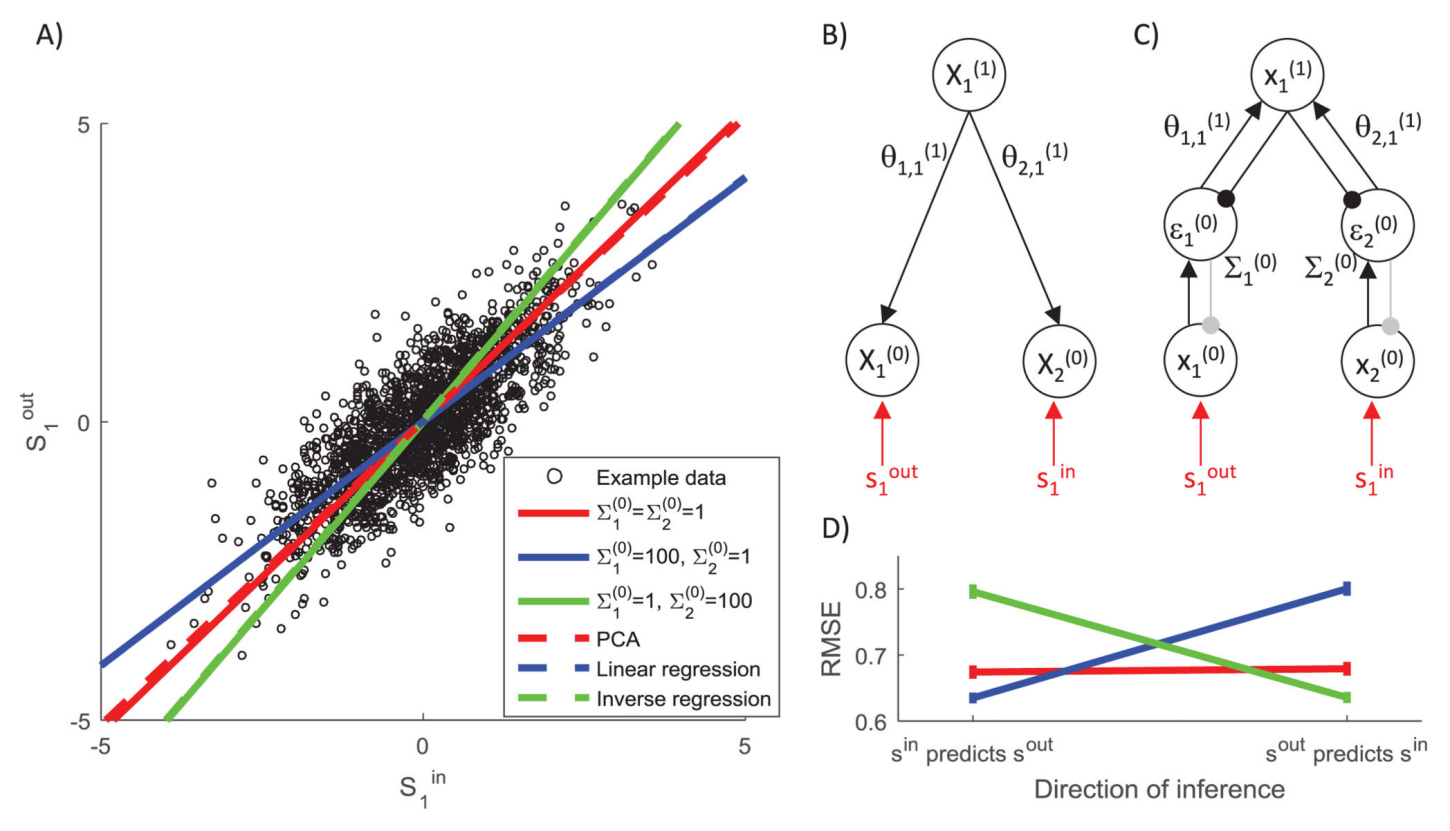
The effect of variance associated with different inputs on network predictions. (A) Sample training set composed of 2000 randomly generated samples, such that $s_{1}^{in}=a+b$ and $s_{1}^{out}=a-b$ where *a* ~ 𝒩 (0, 1) and *b* ~ 𝒩 (0,1/9). Lines compare the predictions made by the model with different parameters with predictions of standard algorithms (see the key). (B) Structure of the probabilistic model. (C) Architecture of the simulated predictive coding network. Notation as in Figure 2. Connections shown in gray are used if the network predicts the value of the corresponding sample. (D) Root mean squared error (RMSE) of the models with different parameters (see the key in panel A) trained on data as in panel A and tested on a further 100 samples generated from the same distribution. During the training, for each sample the network was allowed to converge to the fixed point as described in the caption of Figure 5 and the weights were modified with learning rate *α* = 1. The entire training and testing procedure was repeated 50 times, and the error bars show the standard error.

To understand the potential benefit of such an architecture over the standard backpropagation, we analyze a simple example of learning the association between one-dimensional samples shown in Figure 7A. Since there is a simple linear relationship (with noise) between the samples in Figure 7A, we will consider predictions generated by a very simple network derived from a probabilistic model shown in Figure 7B. During the training of this network, the samples are provided to the nodes on the lowest level ($x_{1}^{\left(0\right)}=s_{1}^{out}$ and $x_{2}^{\left(0\right)}=s_{1}^{in}$).

For simplicity, we assume a linear dependence of variables on the higher level:

(3.4) $$P(x_{i}^{\left(0\right)}|x_{1}^{\left(1\right)})=𝒩\;(x_{i}^{\left(0\right)};\theta_{i,1}^{\left(1\right)}x_{1}^{\left(1\right)},\Sigma_{i}^{\left(0\right)}).$$

Since the node on the highest level is no longer constrained, we need to specify its prior probability, but for simplicity, we assume an uninformative flat prior $P(x_{1}^{\left(1\right)})=c,$ where *c* is a constant. Since the node on the highest level is unconstrained, the objective function we wish to maximize is the logarithm of the joint probability of all nodes:

(3.5) $$F=\text{ln}\left(P({\bar{x}}^{\left(0\right)},x^{1})\right).$$

Ignoring constant terms, this function has an analogous form as in equation 2.15:

(3.6) $$F=-\frac{1}{2}\sum_{i=1}^{n^{\left(0\right)}}\frac{{\left(x_{i}^{\left(0\right)}-\theta_{i,1}^{\left(1\right)}x_{1}^{\left(1\right)}\right)}^{2}}{\Sigma_{i}^{\left(0\right)}}.$$

During training, the nodes on the lowest level are fixed, and the node on the top level is updated proportionally to the derivative of *F*, analogous to the models discussed previously:

(3.7) $${\dot{x}}_{1}^{\left(1\right)}=\sum_{i=1}^{n^{\left(0\right)}}\varepsilon_{i}^{\left(0\right)}\theta_{i,1}^{\left(1\right)}.$$

As before, such computation can be implemented in the simple network shown in Figure 7C. After the nodes converge, the weights are modified to maximize *F*, which here is simply $\Delta \theta_{i,1}^{\left(1\right)}∼\varepsilon_{i}^{\left(0\right)}x_{1}^{\left(1\right)}.$

During testing, we only set $x_{2}^{\left(0\right)}=s_{1}^{in}$ and let both nodes $x_{1}^{\left(1\right)}$ and $x_{1}^{\left(0\right)}$ to be updated to maximize *F*—the node on the top level evolves according to equation 3.7, while at the bottom level, ${\dot{x}}_{i}^{\left(0\right)}=\varepsilon_{i}^{\left(0\right)}.$

This simple linear dependence could be captured by using a predictive coding network without a hidden layer and just by learning the means and covariance matrix, that is, $P(\overset{¯}{x})=𝒩(\overset{¯}{x};\overset{¯}{\mu },\Sigma )$, where $\overset{¯}{\mu }$ is the mean and **Σ** the covariance matrix. However, we use a hidden layer to show the more general network that could learn more complicated relationships if nonlinear activation functions are used.

The solid lines in Figure 7A show the values predicted by the model (i.e., $x_{1}^{*(0)})$ after providing different inputs (i.e., $x_{2}^{\left(0\right)}=s_{1}^{in}$), and different colors correspond to different noise parameters. When equal noise is assumed in input and output (red line), the network learns the probabilistic model that explains the most variance in the data, so the model learns the direction in which the data are most spread out. This direction is the same as the first principal component shown in the dashed red line (any difference between the two lines is due to the iterative nature of learning in the predictive coding model).

When the noise parameter at the node receiving output samples is large (the blue line in Figure 7A), the dynamics of the network will lead to the node at the top level converging to the input sample (i.e., $x_{1}^{*(1)}\approx s_{1}^{in}$). Given the analysis presented earlier, the model converges then to the backpropagation algorithm, which in the case of linear *f* (*x*) simply corresponds to linear regression, shown by the dashed blue line.

Conversely, when the noise at the node receiving input samples is large (the green line in Figure 7A), the dynamics of the network will lead to the node at the top level converging to the output sample (i.e., $x_{1}^{*(1)}\approx s_{1}^{out}$). The network in this case will learn to predict the input sample on the basis of the output sample. Hence, its predictions correspond to that obtained by finding linear regression in inverse direction (i.e., the linear regression predicting *s^in^* on the basis of *s^out^*), shown by the dashed green line.

Different predictions of the models with different noise parameters will lead to different amounts of error when tested, which are shown in the left part of Figure 7D (labeled “*s^in^* predicts *s^out^*”). The network approximating the backpropagation algorithm is the most accurate, as the backpropagation algorithm explicitly minimizes the error in predicting output samples. Next in accuracy is the network with equal noise on both input and output, followed by the model approximating inverse regression.

Due to the flexible structure of the predictive coding network, we can also test how well it is able to infer the likely value of input sample *s^in^* on the basis of the output sample *s^out^*. In order to test it, we provide the trained network with the output sample $\left(x_{1}^{\left(0\right)}=s_{1}^{out}\right)$ and let both nodes $x_{1}^{\left(1\right)}$ and $x_{2}^{\left(0\right)}$ be updated. The value $x_{2}^{*(0)}$ to which the node corresponding to the input converged is the network’s inferred value of the input. We compared these values with actual *s^in^* in the testing examples, and the resulting root mean squared errors are shown in the right part of Figure 7D (labeled “*s^out^* predicts *s^in^*”). This time, the model approximating the inverse regression is most accurate.

Figure 7D illustrates that when noise is present in the data, there is a trade-off between the accuracy of inference in the two directions. Nevertheless, the predictive coding network with equal noise parameters for inputs and outputs is predicting relatively well in both directions, being just slightly less accurate than the optimal algorithm for the given direction.

It is also important to emphasize that the models we analyzed in this section generate different predictions only because the training samples are noisy. If the amount of noise were reduced, the models’ predictions would become more and more similar (and their accuracy would increase). This parallels the property discussed earlier that the closer the predictive coding models predict all samples in the training set, the closer their computation to ANNs with backpropagation algorithm.

The networks in the cortex are likely to be nonlinear and include multiple layers, but predictive coding models with corresponding architectures are still likely to retain the key properties outlined above. Namely, they would allow learning bidirectional associations between inputs and outputs, and if the mapping between the inputs and outputs could be perfectly represented by the model, the networks could be able to learn them and make accurate predictions.

## 4 Discussion

In this letter, we have proposed how the predictive coding models can be used for supervised learning. We showed that they perform the same computation as ANNs in the prediction mode, and weight modification in the learning mode has a similar form as for the backpropagation algorithm. Furthermore, in the limit of parameters describing the noise in the layer where output training samples are provided, the learning rule in the predictive coding model converges to that for the backpropagation algorithm.

### 4.1 Biological Plausibility of the Predictive Coding Model

In this section we discuss various aspects of the predictive coding model that require consideration or future work to demonstrate the biological plausibility of the model.

In the first model we presented (see section 2.2) and in the simulations of handwritten digit recognition, the inputs and outputs corresponded to layers different from the traditional predictive coding model (Rao & Ballard, 1999), where the sensory inputs are presented to layer *l* = 0 while the higher layers extract underlying features. However, supervised learning in a biological context would often involve presenting the stimuli to be associated (e.g., image of a letter, and a sound) to sensory neurons in different modalities and thus would involve the network from “input modality” via the higher associative cortex to the “output modality.” We focused in this letter on analyzing a part of this network from the higher associative cortex to the output modality, and thus we presented *s^out^* to nodes at layer *l* = 0. We did this only for this case because it is easy to show analytically the relationship between predictive coding and ANNs. Nevertheless, we would expect the predictive coding network to also perform supervised learning when *s^in^* is presented to layer 0, while *s^out^* to layer *l*_max_, because the model minimizes the errors between predictions of adjacent levels so it learns the relationships between the variables on adjacent levels. It would be an interesting direction for future work to compare the performance of the predictive coding networks with input and outputs presented to different layers.

In section 3.3, we briefly considered a more realistic architecture involving both modalities represented on the lowest-level layers. Such an architecture would allow for a combination of supervised and unsupervised learning. If one no longer has a flat prior on the hidden node but a gaussian prior (so as to specify a generative model), then each arm could be trained separately in an unsupervised manner, while the whole network could also be trained together. Consider now that the input to one of the arms is an image and the input at the other arm is the classification. It would be interesting to investigate if the image arm could be pretrained separately in an unsupervised manner alone and if this would speed up learning of the classification.

We now consider the model in the context of the plausibility criteria stated in section 1. The first two criteria of local computation and plasticity are naturally satisfied in a linear version of the model (with *f* (*x*) = *x*), and we discussed possible neural implementation of nonlinearities in the model (see Figure 3). In that implementation, some of the neurons have a linear activation curve (like the value node $x_{1}^{\left(2\right)}$ in Figure 3) and others are nonlinear (like the node $f(x_{1}^{\left(2\right)})$), which is consistent with the variability of the firing-input relationship (or f-I curve) observed in biological neurons (Bogacz, Moraud, Abdi, Magill, & Baufreton, 2016).

The third criterion of minimal external control is also satisfied by the model, as it performs computations autonomously given input and outputs. The model can also autonomously “recognize” when the weights should be updated, because this should happen once the nodes converged to an equilibrium and have stable activity. This simple rule would result in weight update in the learning mode, but no weight change in the prediction mode, because then the prediction error nodes have activity equal to 0, so the weight change (see equation 2.19) is also 0. Nevertheless, without a global control signal, each synapse could detect only if the two neurons it connects have converged. It will be important to investigate if such a local decision of convergence is sufficient for good learning.

The fourth criterion of plausible architecture is more challenging for the predictive coding model. First, the model includes special one-to-one connections between variable nodes $\left(x_{i}^{\left(l\right)}\right)$ and the corresponding prediction error nodes $(\varepsilon_{i}^{\left(l\right)}),$ while there is no evidence for such special pairing of neurons in the cortex. It would be interesting to investigate if the predictive coding model would still work if these one-to-one connections were replaced by distributed ones. Second, the mathematical formulation of the predictive coding model requires symmetric weights in the recurrent network, while there is no evidence for such a strong symmetry in cortex. However, our preliminary simulations suggest that symmetric weights are not necessary for good performance of predictive coding network (as we will discuss in a forthcoming paper). Third, the error nodes can be either positive or negative, while biological neurons cannot have negative activity. Since the error neurons are linear neurons and we know that rectified linear neurons exist in biology (Bogacz et al., 2016), a possible way we can approximate a purely linear neuron in the model with a biological rectified linear neuron is if we associate zero activity in the model with the baseline firing rate of a biological neuron. Nevertheless, such an approximation would require the neurons to have a high average firing rate, so that they rarely produce a firing rate close to 0, and thus rarely become nonlinear. Although the interneurons in the cortex often have higher average firing rates, the pyramidal neurons typically do not (Mizuseki & Buzsáki, 2013). It will be important to map the nodes in the model on specific populations in the cortex and test if the model can perform efficient computation with realistic assumptions about the mean firing rates of biological neurons.

Nevertheless, predictive coding is an appealing framework for modeling cortical networks, as it naturally describes a hierarchical organization consistent with those of cortical areas (Friston, 2003). Furthermore, responses of some cortical neurons resemble those of prediction error nodes, as they show a decrease in response to repeated stimuli (Brown & Aggleton, 2001; Miller & Desimone, 1993) and an increase in activity to unlikely stimuli (Bell, Summerfield, Morin, Malecek, & Ungerleider, 2016). Additionally, neurons recently reported in the primary visual cortex respond to a mismatch between actual and predicted visual input (Fiser et al., 2016; Zmarz & Keller, 2016).

### 4.2 Does the Brain Implement Backprop?

This letter shows that a predictive coding network converges to backpropagation in a certain limit of parameters. However, it is important to add that this convergence is more of a theoretical result, as it occurs in a limit where the activity of error nodes becomes close to 0. Thus, it is unclear if real neurons encoding information in spikes could reliably encode the prediction error. Nevertheless, the conditions under which the predictive coding model converges to the backpropagation algorithm are theoretically useful, as they provide alternate probabilistic interpretations of the backpropagation algorithm. This allows a comparison of the assumptions made by the backpropagation algorithm with the probabilistic structure of learning tasks and questions whether setting the parameters of the predictive coding models to those approximating backpropagation is the most suitable choice for solving real-world problems that animals face.

First, the predictive coding model corresponding to backpropagation assumes that output samples are generated from a probabilistic model with multiple layers of random variables, but most of the noise is added only at the level of output samples (i.e., $\Sigma_{i}^{\left(0\right)}>>\Sigma_{i}^{\left(l>0\right)}$). By contrast, probabilistic models corresponding to most of real-world data sets have variability entering on multiple levels. For example, if we consider classification of images of letters, the variability is present in both high-level features like length or angle of individual strokes and low-level features like the colors of pixels.

Second, the predictive coding model corresponding to backpropagation assumes a layered structure of the probabilistic model. By contrast, probabilistic models corresponding to many problems may have other structures. For example, in the task from section 1 of a child learning the sounds of the letters, the noise or variability is present in both the visual and auditory stimuli. Thus, this task could be described by a probabilistic model including a higher-level variable corresponding to a letter, which determines both the mean visual input perceived by a child and the sound made by the parent. Thus, the predictive coding networks with parameters that do not implement the backpropagation algorithm exactly may be more suited for solving the learning tasks that animals and humans face.

In summary, the analysis suggests that it is unlikely that brain networks implement the backpropagation algorithm exactly. Instead, it seems more probable that cortical networks perform computations similar to those of a predictive coding network without any variance parameters dominating any others. These networks would be able to learn relationships between modalities in both directions and flexibly learn probabilistic models well describing observed stimuli and the associations between them.

### 4.3 Previous Work on Approximation of the Backpropagation Algorithm

As we mentioned in section 1, other models have been developed describing how the backpropagation algorithm could be approximated in a biological neural network. We now review these models, relate them to the four criteria stated in section 1, and compare them with the predictive coding model.

O’Reilly (1998) considered a modified ANN that also includes feedback weights between layers that are equal to feedforward weights. In this modified ANN, the output of hidden nodes in the equilibrium is given by

(4.1) $$o_{i}^{\left(l\right)}=f\left(\sum_{j=1}^{n^{\left(l+1\right)}}w_{i,j}^{\left(l+1\right)}o_{j}^{\left(l+1\right)}+\sum_{j=1}^{n^{\left(l-1\right)}}w_{j,i}^{\left(l\right)}o_{j}^{\left(l-1\right)}\right),$$

and the output of the output nodes satisfies in equilibrium the same condition as for the standard ANN (an equation similar to the one above but including just the first summation). It has been demonstrated that the weight change minimizing the error of this network can be well approximated by the following update (O’Reilly, 1998):

(4.2) $$\Delta w_{i,j}^{\left(l\right)}∼o_{i}^{(l-1),train}o_{j}^{(l),train}-o_{i}^{(l-1),pred}o_{i}^{(l),pred}.$$

This is the contrastive Hebbian learning weight update rule (Ackley et al., 1985). In equation 4.2, $o_{j}^{(l),pred}$ denotes the output of the nodes in the prediction phase, when the input nodes are set to $o_{j}^{\left(l_{\text{max}}\right)}=s_{j}^{in}$ and all the other nodes are updated as described above, while $o_{j}^{(l),train}$ denotes the output in the training phase when, in addition, the output nodes are set to $y_{j}^{\left(0\right)}=s_{j}^{out}$ and the hidden nodes satisfy equation 4.1. Thus, according to the plasticity rule, each synapse needs to be updated twice—once after the network settles to equilibrium during prediction and once after the network settles following the presentation of the desired output sample. Each of these two updates relies just on local plasticity, but they have the opposite sign. Thus, the synapses on all levels of hierarchy need “to be aware” of the presence of *s^out^* on the output and use Hebbian or anti-Hebbian plasticity accordingly. Although it has been proposed how such plasticity could be implemented (O’Reilly, 1998), it is not known if cortical synapses can perform such form of plasticity.

In the above GeneRec model, the error terms *δ* are not explicitly represented in neural activity, and instead the weight change based on errors is decomposed into a difference of two weight modifications: one based on target value and one based on predicted value. By contrast, the predictive coding model includes additional nodes explicitly representing error and, thanks to them, has a simpler plasticity rule involving just a single Hebbian modification. A potential advantage of such a single modification is robustness to uncertainty about the presence of *s^out^* because no mistaken weight updates can be made when *s^out^* is not present.

Bengio and colleagues (Bengio, 2014; Bengio et al., 2015) considered how the backpropagation algorithm can be approximated in a hierarchical network of autoencoders that learn to predict their own inputs. The general frameworks of autoencoders and predictive coding are closely related, as both of the networks, which include feedforward and feedback connections, learn to predict activity on lower levels from the representation on the higher levels. This work (Bengio, 2014; Bengio et al., 2015) includes many interesting results, such as improvement of learning due to the addition of noise to the system. However, it was not described how it is mapped on a network of simple nodes performing local computation. There is a discussion of a possible plasticity rule at the end of Bengio (2014) that has a similar form as equation 4.2 of the GeneRec model.

Bengio and colleagues (Scellier & Bengio, 2016; Bengio & Fischer, 2015) introduce another interesting approximation to implement backpropagation in biological neural networks. It has some similarities to the model presented here in that it minimizes an energy function. However, like contrastive Hebbian learning, it operates in two phases, a positive and a negative phase, where weights are updated from information obtained from each phase. The weights are changed following a differential equation update starting at the end of the negative phase and until convergence of the positive phase. Learning must be inhibited during the negative phase, which would require a global signal. This model also achieves good results on the MNIST data set.

Lillicrap et al. (2016) focused on addressing the requirement of the backpropagation algorithm that the error terms need to be transmitted backward through exactly the same weights that are used to transmit information feedforward. Remarkably, they have shown that even if random weights are used to transmit the errors backward, the model can still learn efficiently. Their model requires external control over nodes to route information differentially during training and testing. Furthermore, we note that the requirement of symmetric weights between the layers can be enforced by using symmetric learning rules like those proposed in GeneRec and predictive coding models. Equally, we will show in a future paper that the symmetric requirement is not actually necessary in the predictive coding model.

Balduzzi et al. (2014) showed that efficient learning may be achieved by a network that receives a global error signal and in which synaptic weight modification depends jointly on the error and the terms describing the influence of each neuron of final error. However, it is not specified in this work how these influence terms could be computed in a way satisfying the criteria stated in section 1.

Finally, it is worth pointing out that previous papers have shown that certain models perform similar computations as ANNs or that they approximate the backpropagation algorithm, while in this letter, we show, for the first time, that a biologically plausible algorithm may actually converge to backpropagation. Although this convergence in the limit is more of a theoretical result, it provides a mean to clarify the computational relationship between the proposed model and backpropagation, as described above.

### 4.4 Relationship to Experimental Data

We hope that the proposed extension of the predictive coding framework to supervised learning will make it easier to test this framework experimentally. The model predicts that in a supervised learning task, like learning sounds associated with shapes, the activity after feedback, proportional to the error made by a participant, should be seen not only in auditory areas but also visual and associative areas. In such experiments, the model can be used to estimate prediction errors, and one could analyze precisely which cortical regions or layers have activity correlated with model variables. Inspection of the neural activity could in turn refine the predictive coding models, so they better reflect information processing in cortical circuits.

The proposed predictive coding models are still quite abstract, and it is important to investigate if different linear or nonlinear nodes can be mapped on particular anatomically defined neurons within a cortical microcircuit (Bastos et al., 2012). Iterative refinements of such mapping on the basis of experimental data (such as f-I curves of these neurons, their connectivity, and activity during learning tasks) may help understand how supervised and unsupervised learning is implemented in the cortex.

Predictive coding has been proposed as a general framework for describing computations in the neocortex (Friston, 2010). It has been shown in the past how networks in the predictive coding framework can perform unsupervised learning, attentional modulations, and action selection (Rao & Ballard, 1999; Feldman & Friston, 2010; Friston, Daunizeau, Kilner, & Kiebel, 2010). Here we add to this list supervised learning, and associative memory (as the networks presented here are able to associate patterns of neural activity with each other). It is remarkable that the same basic network structure can perform this variety of the computational tasks, also performed by the neocortex. Furthermore, this network structure can be optimized for different tasks by modifying proportions of synapses among different neurons. For example, the networks considered here for supervised learning did not include connections encoding covariance of random variables, which are useful for certain unsupervised learning tasks (Bogacz, 2017). These properties of the predictive coding networks parallel the organization of the neocortex, where the same cortical structure is present in all cortical areas, differing only in proportions and properties of neurons and synapses in different layers.

## Figures and Tables

**Table 1:** Corresponding and Common Symbols Used in Describing ANNs and Predictive Coding Models.

	Backpropagation	Predictive Coding
Activity of a node (before nonlinearity)	$y_{i}^{\left(l\right)}$	$x_{i}^{\left(l\right)}$
Synaptic weight	$w_{i,j}^{\left(l\right)}$	$\theta_{i,j}^{\left(l\right)}$
Objective function	*E*	*F*
Prediction error	$\delta_{i}^{\left(l\right)}$	$\varepsilon_{i}^{\left(l\right)}$
Activation function	*f*	
Number of neurons in a layer	*n* ^(*l*)^	
Highest index of a layer	*l*_max_	
Input from the training set	$s_{i}^{in}$	
Output from the training set	$s_{i}^{out}$	

## Footnotes

1 As in previous work linking the backpropagation algorithm to probabilistic inference (Rumelhart, Durbin, Golden, & Chauvin, 1995), we consider the output from the network to be $y_{i}^{\left(0\right)}$ rather than $f(y_{i}^{\left(0\right)}),$ as it simplifies the notation of the equivalent probabilistic model. This corresponds to an architecture in which the nodes in the output layer are linear. A predictive coding network approximating an ANN with nonlinear nodes in all layers was derived in a previous version of this letter (Whittington & Bogacz, 2015).
